# Operating envelope of Haber–Bosch process design for power-to-ammonia†

**DOI:** 10.1039/c8ra06821f

**Published:** 2018-10-11

**Authors:** Izzat Iqbal Cheema, Ulrike Krewer

**Affiliations:** Institute of Energy and Process Systems Engineering, TU Braunschweig Braunschweig 38106 Germany u.krewer@tu-braunschweig.de +49 531 3915932 +49 531 3913030; International Max Planck Research School for Advanced Methods in Process and Systems Engineering Magdeburg 39106 Germany; Department of Chemical, Polymer and Composite Materials Engineering, University of Engineering and Technology, Lahore KSK-Campus 39021 Pakistan

## Abstract

The power-to-ammonia concept allows for the production of ammonia, one of the most produced inorganic chemicals, from air, water and (renewable) electricity. However, power-to-ammonia requires flexible operation for use with a directly intermittent renewable energy supply. In this paper, we systematically analyse the operating envelope for steady-state operation of the three bed autothermic Haber–Bosch reactor system for power-to-ammonia by pseudo-homogeneous model. Operational flexibilities of process variables, hydrogen intake and ammonia production flexibilities are analysed, along with maximum and minimum possible changes in recycle load and recycle to feed ratio for the following process variables: reactor pressure, inert gas percentage in synthesis loop, NH_3_ concentration, H_2_-to-N_2_ ratio, total flow rate and feed temperature. Among the six process variables, inert gas fraction and H_2_-to-N_2_ ratio provided very high flexibilities, *ca.* 255% operational flexibility for Ar, up to 51 to 67% flexibility in hydrogen intake, and up to 73% reduction and 24% enhancement in ammonia production. However, a decrease in ammonia production by H_2_-to-N_2_ ratio significantly increases recycle load. Besides inert gas fraction and H_2_-to-N_2_ ratio, the total mass feed flow rate is also significant for minimum hydrogen intake and ammonia production.

## Abstract 2

The Haber–Bosch process is viable for power-to-ammonia, as it can be operated for a wide range of the operating envelope while maintaining the process variables' operational, hydrogen feed intake and ammonia production flexibilities.

## Graphical abstract

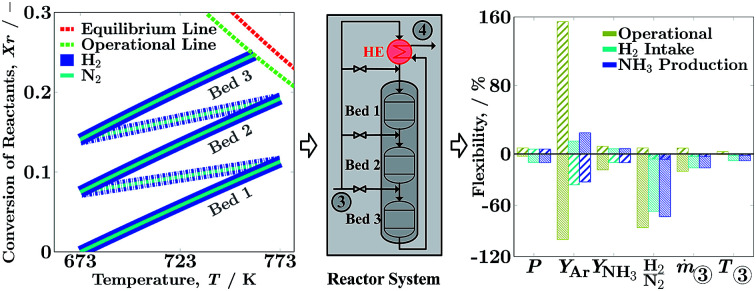

## 1 Introduction

Ammonia is the second most produced industrial chemical, and the production process has been intensively developed over a period of one century. Ammonia is used as raw material for the production of various nitrogen compounds, including nitric acid, and a variety of fertilisers and polymers. Also, ammonia is used as refrigerant and neutraliser for NO_*x*_ emission from fuel combustion.^1^ Moreover, ammonia has been tested and applied as fuel in compression ignition engines,^2–4^ spark ignition engines,^5–7^ gas turbines^8–10^ and fuel cells^11,12^ over a period of time. Despite its toxicity, ammonia has an excellent safety record in the fertiliser industry and a well established transportation network.^13,14^ Thus, an ammonia economy would be low in cost and easier to apply than hydrogen in the energy sector.

Currently, about 1.6% of fossil fuels, such as coal and natural gas, is used worldwide for the manufacturing of ammonia.^1^ The classical production method, the Haber–Bosch process, relies heavily on natural gas,^15^ whereas ammonia has also the capability of being produced from renewable energy sources *e.g.* solar^16^ and wind.^17–19^ Fuhrmann *et al.*^19^ reviewed the classical Haber–Bosch process and alternative electro-chemical ammonia production concepts. They also discussed the potential for dynamic or flexible operation of the developed Haber–Bosch process concept, and as such, its ability to flexibly store excess renewable energy. With the growth of renewable energy production, power-to-ammonia and ammonia-to-power has garnered world-wide interest. The current activities related to renewable ammonia in the U.S., Europe and Japan are comprehensively highlighted by Pfromm.^20^

Power-to-ammonia will rely on H_2_ production by splitting of water *via* electrolysis, where N_2_ will be separated from air *e.g.* by pressure swing adsorption and cryogenic distillation.^19^ The Haber–Bosch (HB) ammonia synthesis loop itself has shown to be similar to the conventional one.^16,18,19^ For the power-to-ammonia concept *via* Haber–Bosch synthesis loop, a technology readiness level of 6 has already been accomplished by Proton Ventures BV, The Netherlands.^16^ The first pilot plant has been operational at West Central Research and Outreach Center, Morris, Minnesota, USA since 2013^18^ and the second demonstrator became operational in June 2018 at Science & Technology Facilities Council's, Rutherford Appleton Laboratory, Oxfordshire.^21^ The operation of power-to-ammonia plant by West Central Research and Outreach Center, Morris, Minnesota, USA has only been studied at steady state, not dynamically. The efficiency of power-to-ammonia is estimated between 50 and 60%, including hydrogen and nitrogen production,^22^ which is lower than from the latest classical Haber–Bosch ammonia production plants *i.e.* between 60 and 64%.^23^ This is mainly due to higher energy requirements and energy losses in production of H_2_ from electrolysis of water by atmospheric alkaline, high pressure alkaline (16 bar) or proton exchange membrane electrolysis cells.^22^

Simulations of the power-to-ammonia process were carried out for a system consisting of electrolyser, cryogenic separation and Haber–Bosch by Sánchez & Martín,^24^ while low temperature and high temperature electrolyser, pressure swing adsorption and Haber–Bosch were presented by Cinti *et al.*^25^ Cinti *et al.* analysed energy performances along with electricity consumption for every individual section. For the Haber–Bosch loop, thermodynamic equilibrium is considered instead of a kinetic approach, which is suitable for design-based analysis only. On the other hand, Sánchez & Martín carried out complete system simulation and operation optimisation, including a kinetic approach for Haber–Bosch synthesis reactor. Even so, they didn't consider an autothermic ammonia synthesis reactor, which is of high interest for realising stand-alone power-to-ammonia plants. The synthesis reaction (see eqn (1)) is highly exothermic and equilibrium driven. Despite this fact, the reaction may be carried out in an autothermal synthesis reactor system.^1^ So far, the question of how much an autothermal Haber–Bosch reactor system can be operated flexibly outside its standard conditions, is of crucial relevance for the power-to-ammonia concept, but has not been addressed. An alternative approach is to realise constant NH_3_ production for the power-to-ammonia process proposed, mainly with help of the uninterrupted reactants supply. The uninterrupted supply of the reactants is maintained either by the continuous production of reactants with the help of non-stop supply of electricity or *via* producing excess amount of reactants which are stored during surplus energy and which are used during shortfall time.^26^ However, storing H_2_ reactant in bulk over a day can be up to three times more expensive than ammonia; in fact an ammonia storage tank is the cheapest and largest energy storage battery (greater than 100 GW h).^26,27^ Therefore, for answering the question raised above, knowledge of the operating envelope is essential, in case the Haber–Bosch process should be used for on-demand, flexible production of ammonia. In this work we present design and off-design analysis of the ammonia synthesis reactor system, and we will consider both, kinetic and autothermic approaches. The following section gives an analysis on the exact challenges a flexible Haber–Bosch process faces, which then will be analysed using modelling in later sections.1
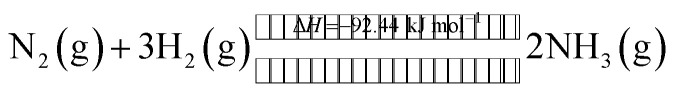


### 1.1 Haber–Bosch process

**Fig. 1:**
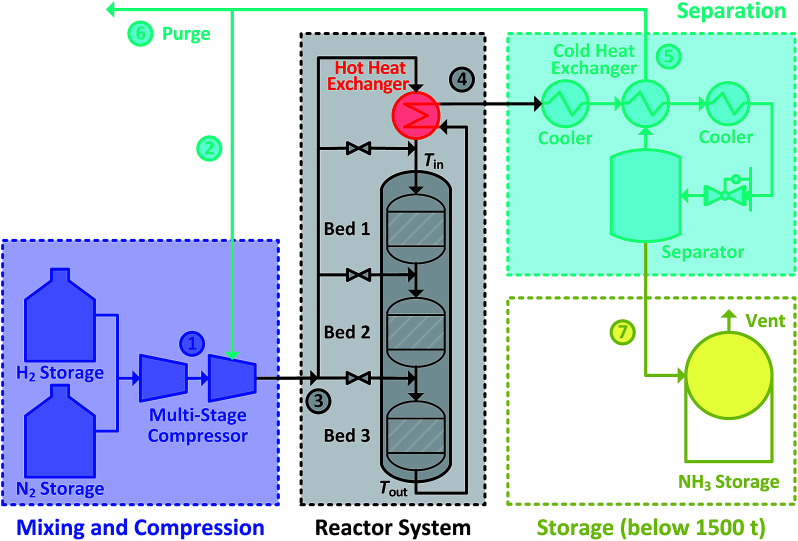
Ammonia synthesis loop with small quantity ammonia storage for power-to-ammonia.

The Haber–Bosch ammonia synthesis loop for producing NH_3_ consists of mixing and compression units, synthesis reactor system, a trail of heat exchangers and coolers, a separator, a recycle loop and a storage unit. Altogether, it can be divided into four subsections, as shown in Fig. 1. The system design of the ammonia synthesis reactor poses a challenge due to the harsh reactor requirements of high inlet temperature to achieve high reaction rate and simultaneously, low outlet temperature to achieve a high equilibrium conversion.^28^ Furthermore, a high reactant conversion should be achieved despite constraints due to equilibrium conversion. This is accomplished through the use of several catalyst beds in series.^29^ The usual operational envelope ranges are: pressure of 150 to 300 bar, temperature of 623 to 773 K, H_2_-to-N_2_ molar ratios of 2 : 1 to 3 : 1 and inert gas content from 0 to 15 mol%.^1^ The operational envelopes mentioned above for carrying out the ammonia synthesis reaction are quite general, and vary greatly. However, Haber–Bosch process plants have some constraints imposed due to design^30,31^ and operation limitations,^32^ which originate from requirements of autothermic operation of the reactor system, catalyst type, feed content and composition. Therefore, the operating envelope needs to be determined and customised with respect to the process plant. Furthermore, due to low conversion (25 to 35%), un-reacted reactants need to be separated and recycled back.^1^ Therefore, the recycled reactants flow rate (recycle load) is several times higher from the feed flow rate. In the power-to-ammonia synthesis loop, the only inert gas is argon,^22^ originating from the air separation unit, along with the N_2_ used as a reactant. In the conventional process, inert gases are CH_4_ and Ar.^1^ Concentration of Ar in the synthesis loop is controlled by purging a small amount of gas from the recycle stream.^22^

During the power-to-ammonia pilot plant operation at Morris, Minnesota, USA it was determined that the production of ammonia is controlled by three bottlenecks in the ammonia synthesis loop: catalytic reaction, NH_3_ separation by condensation and recycling of unreacted reactants. Among these production bottlenecks, catalytic reaction has at least three times higher influence than the others.^18^ In ammonia synthesis reactor system, the temperature conditions for inlet and outlet are managed by exchanging heat between outlet and inlet streams. The heat of reaction is itself sufficient for maintaining the temperature level in the reactor system, allowing the process to be operated autothermically, see Fig. 1. However, this requires careful heat management in the reactor system, particularly between inlet and outlet streams. If the inlet stream is not sufficiently heated, the rate of reaction will drop and will lead to lower outlet temperature, which results in lowering inlet temperature and eventually the reaction will stop completely.^33^ Therefore, the analysis and careful operation of the ammonia synthesis reactor system in an ammonia synthesis loop carries great importance and is the focus of this work.

Much of the work regarding the ammonia synthesis reactor system revolved around an incident that occurred in an industrial ammonia fixed-bed synthesis reactor in Germany in 1989.^34^ Multiplicity of periodic behaviour and stability analysis of ammonia reactor systems are repeatedly mentioned in the literature.^34–38^ But much of the work only highlighted the effect of reactor operational pressure, inlet temperature and feed temperature, and did not consider feed flow rate and feed composition *e.g.* H_2_-to-N_2_ molar ratio, NH_3_ and inert gas concentration. These variables, though, would be essential to manipulate during operation of a flexible power-to-ammonia system. Morud and Skogestad in 1998 analysed the Haber–Bosch process with a pseudo-homogeneous dynamic model for a three catalyst bed reactor system and a static model for a counter current heat exchanger,^34^ Mancusi *et al.* in 2000, 2001 and 2009 analysed the same process with a heterogeneous model and concluded substantial qualitative agreement with the pseudo-homogeneous results *e.g.* shutdown pressure and feed temperature for the reactor system was more than the pseudo-homogeneous by about 18.57 bar^36^ and 20 K.^37^ Azarhoosh *et al.*^39^ also considered a one-dimensional heterogeneous model, and compared results with the real plant and had difference of up to 13.5 K in the catalyst bed. In addition, they also optimised the synthesis reactor for maximum ammonia production by adjusting input temperature, total feed flow rate and operating pressure. Farivar & Ebrahim^40^ extended this work by using a two-dimensional model and a finite volume method. In comparison to their previous work^39^ they reduced the temperature difference to 4 K in the catalyst bed from real plant data. They also analysed the effect of pressure. Furthermore, a simple dynamic model-based stability analysis for a single bed ammonia synthesis reactor and heat exchanger was studied by Rabchuk *et al.*^38^ for a step change of the parameters of pressure, temperature and flow rate. They concluded that a more realistic thermodynamic model needs to be added, and that the reactor system should be extended to a higher number of catalyst beds, corresponding to the real ammonia synthesis reactor system. Among multi-bed reactor systems, *e.g.* two to four catalyst beds, the three bed reactor system is the most efficient and cost effective for NH_3_ production.^31^ The operational and production flexibility for the conventional ammonia synthesis reactor system has not yet been systematically analysed, as the plants are mostly designed for large capacities and the raw material methane is abundantly available and easily storable at highly constant inlet conditions.

The focus of this work is to determine the steady-state operational and production limitations of the ammonia synthesis reactor system and recycle loop, as renewable energy will be only intermittently available for the production of the reactants. H_2_ is the limiting reactant in the power-to-ammonia process, as more than 90% of the energy is consumed during its production. During energy shortage periods, H_2_ production may need to be reduced or even shut down.^22^ Thus, knowing the operational flexibilities of the process variables, H_2_ intake and NH_3_ production flexibilities along with the change in recycle load and recycle to feed ratio is of high relevance and should be analysed. We therefore focus on such an analysis, using the quench based inter-stage cooling three bed ammonia synthesis reactor system, shown in Fig. 1. Special focus is given to guarantee autothermal operation, *i.e.* energy sufficiency without additional heating/cooling. Therefore, we first define the pseudo-homogeneous mathematical model along with the assumptions of the reactor system. Then, the effect of the following process variables is analysed: reactor pressure, inert percentage in synthesis loop, NH_3_ concentration, H_2_-to-N_2_ ratio, total flow rate and inlet temperature of reactor system on the operational envelope, H_2_ intake and NH_3_ production flexibilities, along with change in recycle load and recycle to feed ratio for the reactor system.

## 2 Mathematical model and simulation

Physicochemical modelling is applied to analyse the ammonia synthesis reactor system under steady-state operation. The systematically applied approach subdivides the reactor system into three subsystems *i.e.* heat exchanger, catalyst beds and mixers. The processes taking place within the boundaries of each subsystem are distinguishable physically and/or chemically. By combining the individual subsystems, the behaviour of the overall synthesis system can be quantified. First, the simplifying assumptions, along with mathematical models, are presented. These models are then followed by simulation scenarios for identifying operation, H_2_ intake and NH_3_ production flexibilities for the reactor system along with the change in recycle load and recycle to feed ratio. To focus on the complex reactor system, the design and operational limitation which may originate from the separation section by the heat exchanger, coolers and the NH_3_ separator to recycle stream has been ignored. Therefore, changes in recycle and recycle to feed ratio are independent of any kind of limitations. The detailed design and construction specifications of the reactor system are not within the scope of this work. Therefore, a pseudo-homogeneous reactor model is adapted and heat losses are ignored, though with this assumption, behaviour of the reactor system remains quite similar to real plant.^36,37,39,41^ Future studies may tailor the separation section to the required flexibility envelope of the Haber–Bosch process.

### 2.1 Subsystems models

In the following, the assumptions and physical equations for the subsystems are given.

#### Heat exchanger

All the fluids in the heat exchanger remain in the gaseous phase and as such no condensation is considered for modelling. Hot gas flows through tube side and cold gas flows through shell side of the heat exchangers.^34–38^ The heat exchange between tube and shell side gas takes place using a combination of counter current and cross flow. The temperature of the gases changes in the axial direction of flow and does not change in its radial direction. Heat of conduction in the axial direction is also negligible.^42^ All thermal properties of the gases and the exchanger wall are constant. No heat losses occur to the surroundings due to external insulation, *i.e.* the component is adiabatic. Chemical reaction and mass transfer do not take place. Therefore, the system can be described by a steady state energy balance and the feed-effluent heat exchanger is modelled by an *ε*-NTU model^34^ using the effectiveness *ε* as follows:

2 *T*_s_out__ = *εT*_t_in__ + (1 − *ε*)*T*_s_in__

where *T*_s_out__ is the shell side outlet temperature and *T*_t_in__ is the tube side inlet (catalyst bed 3 outlet) temperature, *T*_s_in__ is the shell side inlet temperature, and *ε* is the heat exchanger effectiveness. The *ε* is constant, independent of change in inlet temperature and generally lies within the range 0.4 to 0.8 depending on the configuration of heat exchanger. In context to Fig. 1, the streams of the heat exchanger will be *T*_s_out__ = *T*_in_, *T*_t_in__ = *T*_out_ and *T*_s_in__ = *T*_③_. The *ε*-NTU model has the advantage over conventional methods as it does not require evaluation of mean temperature differences and detailed design of the heat exchanger. The *ε*-NTU model is also suitable for solving off-design heat exchanger problems.^43^ The thermal effectiveness (eqn S1†) for shell and tube heat exchanger, along with specifications (Table S1†) are given in ESI.†

#### Catalyst bed

The heart of an ammonia synthesis reactor is the isobaric and adiabatic catalyst bed. The reaction takes place at the surface of the catalyst, where nitrogen and hydrogen are consumed, and ammonia is formed in an exothermic reaction. We consider a radial flow catalyst bed, where a gradient of temperature and concentration (or partial pressure) is generated in radial direction. Radial flow catalyst beds also permit the handling of small diameter catalyst particles^1^ with high catalyst efficiency^44^ and almost negligible pressure drop,^45^ therefore we assume isobaric conditions. For fine catalyst particles of size 1.5 to 3 mm, the rate of formation for ammonia can be taken without correction factors such as effectiveness factor and with consideration only for convective driving forces for transport of mass and heat between the flowing gases and catalyst.^44^ Further, the temperature gradient Δ*T* inside the catalyst pellet is negligible, as high thermal conductivity magnetite Fe_3_O_4_ catalyst^46^ is assumed. Therefore, heat transfer resistance between pellet and gas is also neglected. The steady state material and energy balance for the fine catalyst particles in catalyst beds are shown in ^eqn (3)^ and ^(4)^, respectively:3
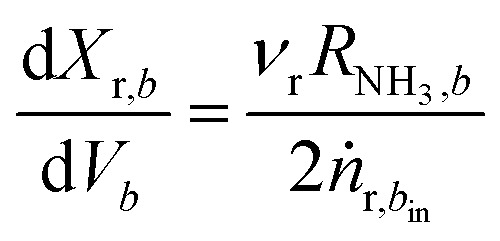
4
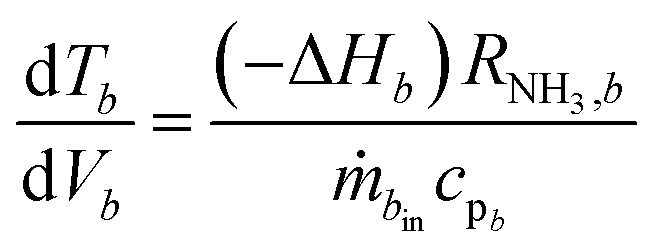
where subscript r ∈ {N_2_ or H_2_} refers to reactants and *b* ∈ {1,2,3} to the three catalyst beds. *ν* is the stoichiometric coefficient, *X* is fractional conversion of reactant, *V* is the volume of the catalyst bed, *R*_NH_3__ is the reaction rate, *ṅ* is the initial molar flow rate of reactant, *T* temperature of reacting mixture, Δ*H* is the heat of reaction, *C*_p_ is the specific heat of reacting mixture and *ṁ* is the total mass flow rate of the reacting mixture. We have considered the conversion differential equation for both reactants, instead of just one reactant, as during the analysis of the operational envelope for H_2_-to-N_2_ ratio we will be shifting limiting reactant between N_2_ and H_2_, which also requires one to change the differential equation. By using reactant conversion, the molar fractions of components are calculated by using eqn S4 to S7, see ESI.†

The rate of reaction is calculated by a modified form of the Temkin equation,^47^ developed in 1968 by Dyson & Simon.^44^ The activities are considered instead of partial pressures, as follows:5
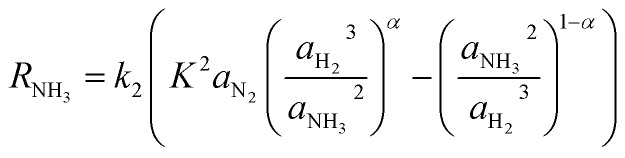
where *a*_N_2__, *a*_H_2__, *a*_NH_3__, *k*_2_, *K* and *α* are activity coefficients for nitrogen, hydrogen and ammonia (eqn S8 to S11, ESI†), constant for reverse reaction (eqn S12, ESI†), equilibrium constant of reaction (eqn S13, ESI†) and constant (Table S2, ESI†), respectively. Also, the equations used for calculating specific heat *C*_p_ (eqn S15 to S17†) and heat of reaction Δ*H* (eqn S18†) are stated in the ESI.†

#### Mixer

The mixing of gases in the mixer is assumed to be ideal and instantaneous. The heat of mixing is neglected, as components do not interact strongly with each other.^48^ Also, pressure remains constant, as isobaric conditions are assumed in overall reactor system. The steady state material and energy balance for the adiabatic mixer are used as follows for calculating the reactant conversion and temperature after quenching:6
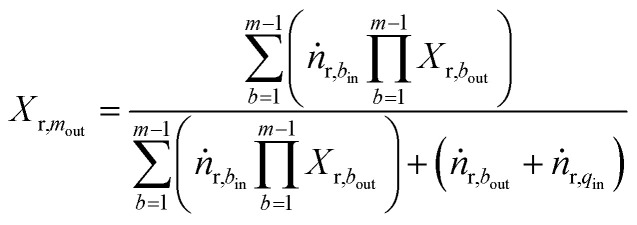
7
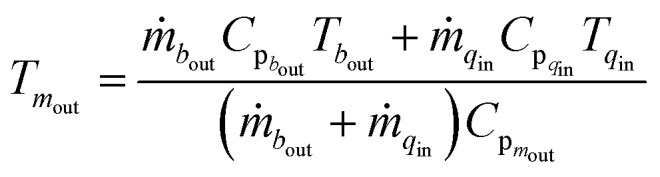


We have considered only the mixers between the catalyst bed in operation *i.e.* mixer 2 and 3. Therefore subscript *q* ∈ {2,3} refers to quench stream, *m* ∈ {2,3} refers to mixers and *b* ∈ {1,2} refers to beds.

#### Flexibility

The equations used for calculating the material balance of streams ① to ⑦ mentioned in Fig. 1 for the ammonia synthesis loop are given in ESI.† The process variables operational flexibility, the H_2_ intake and the NH_3_ production flexibility are defined as a fractional change from the normal values:8
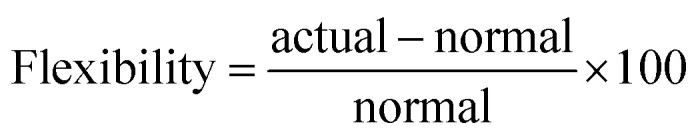


### 2.2 Simulation

The simulation is performed in MATLAB software and a built-in ODE solver (ode45) is used for the implementation of differential equations. For normal operation, the fresh stream ① N_2_ supply with 2 mol% of Ar and pure H_2_ supply from storage is considered in ratio of 3 mol of H_2_ to 1 mol of N_2_. Also, the fresh supply is considered free of impurities like H_2_O and O_2_. After the reactor system, unused reactants are separated from NH_3_ and recycled back with assumption that 27.79 mol% of NH_3_ is carried along with them during normal operation. A concentration of 5 mol% of inert gas is maintained in the reactor system intake stream ③ by purging 0.0241weight fraction of recycle stream ⑤. The initial conditions used are given in Table 1, unless specified separately. The stream numbers are labelled in Fig. 1.

Initial conditions

**Table:** 

Normal (N) operation streams composition/mol%				
Stream	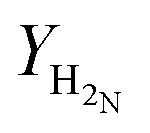	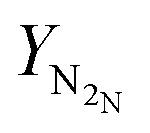	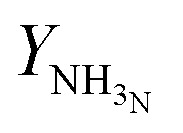	*Y*_Ar_N__
①	74.62	24.88	0.00	0.50
③	68.12	22.71	4.17	5.00

**Table:** 

Inlet & normal (N) operational conditions at reactor system		
*X*_r③_/—	*T*_③N_/K	*P*_N_/bar
0.00	523.00	200.00

The catalyst bed volumes, feed flow rate and quench flow rates *ṁ*_*q*1_, *ṁ*_*q*2_ and *ṁ*_*q*3_ for the given normal operation and feed composition are adjusted by trial and error method for producing 120 kg h^−1^ NH_3_, excluding the 1.11 kg h^−1^ NH_3_ lost in purge gas. For achieving the optimal reactor design volume with the maximum possible reaction rate, inlet temperatures of all catalyst beds are maintained at 673 K and their outlet temperature at 773 K or 90% of the equilibrium temperature. The reactor operation pressure is considered 200 bar which is within the usual operational range mentioned earlier in Section 1.1. With this NH_3_ production capacity, an ammonia-to-power plant is capable of generating 50 MW h per day of energy from *ca.* 3 tons per day of ammonia *via* IC engine of 29% efficiency.^16^ For design only, the reaction is considered to be accomplished when reaching 90% of the equilibrium composition, as for equilibrium conversion operation an infinite amount of reactor space is required.^29^ Also, the reactants and the product present in purge stream were assumed to be lost. The breakdown of the reactor system for each catalyst bed volume and feed flow rate is shown in Table 2.

**Table:** Catalyst bed volumes and normal operation flow rates

Reactor system	Bed 1	Bed 2	Bed 3	Total
*V*/m^3^	0.0075	0.0221	0.0464	0.0760
*ṁ*_*q*_N__/kg h^−1^	—	163.83	177.01	340.84
*ṁ*_*b*_N__/kg h^−1^	321.70	485.53	662.54	662.54

The steady state operating envelope and stability for the autothermic reactor system is investigated with the help of van Heerden plot^33^ for six process variables: reactor pressure, inert concentration, ammonia concentration, H_2_-to-N_2_ ratio, total flow rate and temperature at inlet stream ③ of the reactor system. During the steady-state stability analysis one process variable is changed and the other five process variables are held constant. The plots consist of two different kinds of graphs: the S-shaped heat production curve and the straight-line for heat removal, *e.g.* see Fig. 3. The S-shaped curve shows the relation between temperature of the reactor system bed 1 inlet (*T*_in_) and bed 3 outlet (*T*_out_), rise in temperature is due to exothermic reaction, the straight-line shows the characteristics of heat exchange in the heat exchanger (HE). With help of the heat exchanger, heat is transferred from the bed 3 outlet stream to the bed 1 inlet stream; at steady state operating points, both lines intersect. Under many given operating conditions, multiple steady-states, *i.e.* intersection point of heat production and heat removal lines are obtained. As such, the reactor system can work up to three different steady states characterised by the different temperatures of bed 1 and 3. The lower steady state point and upper steady state point are stable, the upper steady state point is desired for operation due to stability and maximum conversion. The middle steady state point will be unstable: with a minor increase in temperature, the heat of production rises more rapidly than the heat of removal and the temperature will continue increasing until the new point of intersection between heat of production and removal lines is met. For a minor decrease in temperature, the heat of production will continue declining until the point of intersection between heat of production and removal lines met.

## 3 Results and discussions

The results obtained from the model are presented and discussed in this section. First, the reactants fractional conversion and temperature profile along the reactor beds are presented for normal operation. Afterwards, stability analysis is performed for the six process variables to determine operational, H_2_ intake and NH_3_ production flexibilities along with change in recycle and recycle to feed ratio. See Table 3 for results summary. The normal and boundary operation results for each bed inlet and outlet are summarised in Table S4, see ESI.†

**Table:** Reactor system operating envelope and operational flexibility of the process variables, as well as, *ṁ*_H_2_①_ resulting H_2_ intake and *ṁ*_⑦_ NH_3_ production flexibilities along with change in *ṁ*_②_ recycle load and recycle to feed ratio (*ṁ*_②_/*ṁ*_①_)^a^

Process variables	Operating envelope		Flexibility			Change in	
			Operational/%	*ṁ*_H_2_①_/%	*ṁ*_⑦_/%	*ṁ*_②_/%	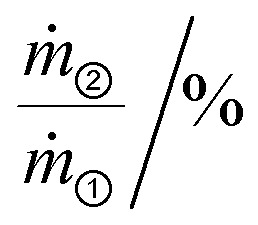
*P*/bar	194.32	Low	−2.84	−9.92	−10.14	+2.49	+13.79
	213.91	High	+6.95	+5.57	+5.72	−1.40	−6.60
*Y*_Ar③_/mol%	0.00	Low	−100.00	+15.00	+24.58	−3.10	−13.77
	12.73	High	+154.60	−36.14	−32.80	+9.08	+70.83
*Y*_NH_3_③_/mol%	3.39	Low	−18.64	+5.99	+6.22	−1.50	−7.07
	4.53	High	+8.85	−10.00	−10.26	+2.51	+13.91
H_2_ : N_2③_/mol of H_2_ : mol of N_2_	1.18 : 2.82	Low	−86.00	−67.15	−73.39	+17.19	+256.80
	3.05 : 0.95	High	+7.01	−5.99	−6.30	+1.62	+8.64
*ṁ*_③_/kg h^−1^	527.78	Low	−20.33	−16.26	−16.16	−21.36	−6.08
	707.61	High	+6.80	−3.00	−3.22	+9.26	+12.65
*T*_③_/K	519.41	Low	−0.68	−7.76	−7.94	+1.95	+10.53
	536.84	High	+2.64	−1.82	−1.86	+0.45	+2.32

a For representing actual limits, rounding off numbers after decimal is not done.

### 3.1 Normal operation

**Fig. 2:**
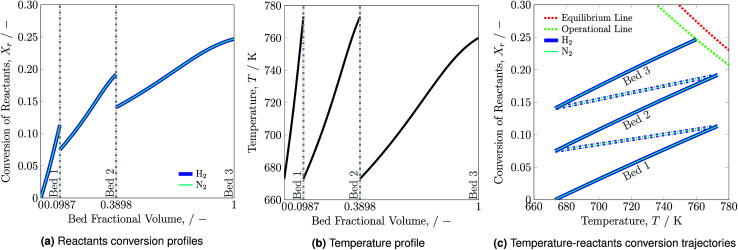
Reactants conversion (a), temperature profiles (b) and temperature-reactants conversion trajectories (c) for the reactor system along the catalyst beds.

Reactants conversion and temperature progression along the catalyst beds are shown in Fig. 2a and b, respectively. The hydrogen and nitrogen conversion profiles overlap, as the reactants' ratio, H_2_-to-N_2_, is stoichiometrically balanced as 3 : 1 (see eqn (1)). Ammonia synthesis is an exothermic reaction that releases heat and therefore the temperature along each bed increases. The rise in reactants conversion and temperature occurs at much higher rate in bed 1 than beds 2 and 3 due to low ammonia content and feed flow rate in bed 1. For accommodating the higher ammonia content and feed flow rate, bed 2 and bed 3 are of larger volume compared to bed 1.

Reactants conversion *versus* temperature and the equilibrium line for the reactor system is presented in Fig. 2c. The solid lines represent temperature and reactants conversion within catalyst beds, whereas dash dotted lines represent temperature and reactant conversion within mixers. The reactor system is operated for the maximum possible reactants conversion and temperature span. For the catalyst bed 3 the 
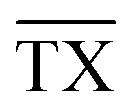
 trajectory touches the operational (OP) line *i.e.* 90% of the equilibrium (EQ) line and reaction is stopped at 760 K as reactor volume was chosen such that 90% conversion may occur to avoid infinite amount of reactor space required for reaching to equilibrium. The effectiveness of heat exchanger *ε* = 0.6329, which is calculated by using eqn (2) for normal operation temperature range. It remains constant during stability analysis of the reactor system and help in determining the intersection temperature. The reactants conversion and temperature from 773 to 673 K within mixers decrease due to quenching of fresh feed. Results summary for normal operation are presented in Table S4, see ESI.†

### 3.2 Operational and production flexibilities

In the following subsection, we analyse the operating envelope, *i.e.* the lower (L) and higher (H) operating points of the autothermic reactor system for the main process variables: reactor pressure, inert concentration, ammonia concentration, H_2_-to-N_2_ ratio, total flow rate and temperature at the inlet of the reactor system. The summary of operating envelope, operational flexibility of the respective process variable, hydrogen intake and ammonia production flexibilities, along with the resulting change in recycle load and recycle to feed ratio is given in Table 3.

**Fig. 3:**
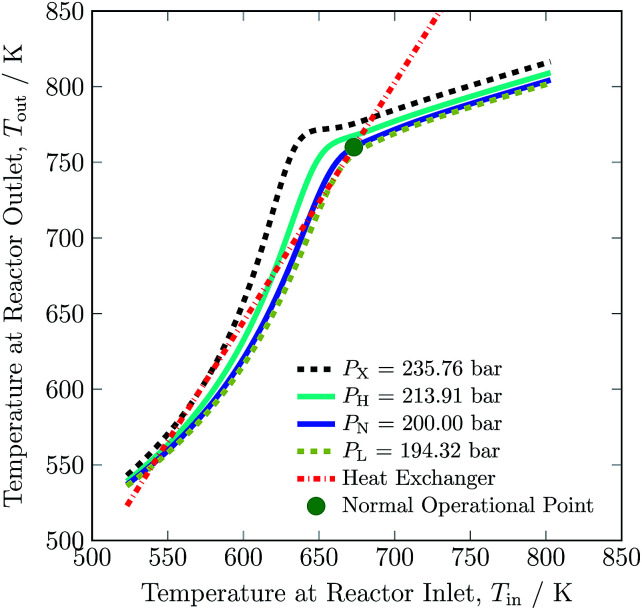
Steady-state characteristics of the reactor system for highest (X), high (H), normal (N) and low (L) operational pressures of the reactor system.

The stability analysis for the reactor pressure is presented in Fig. 3. For the normal (N) reactor operation at 200 bar, it is required that the feed must enter bed 1 at 673 K. For lower temperatures, the reactor will not be able to produce sufficient heat to maintain the reaction, and the inlet temperature at bed 1 would move towards unstable steady state temperature *ca.* 644 K. Further cooling from this point will result in the shut down of reactor system, due to more heat removal than heat production. Likewise, the heat production curve can be moved up and down by changing reactor pressure, until it intersects the heat removal curve at two or one point(s) instead of three points *i.e.* from 194.32 to 235.76 bar or onwards. The increase in pressure increases reactants conversion (see Table S4, ESI†) due to higher reaction rate, thus temperature also increases and the temperature in bed 1 reaches the upper limit of catalyst *i.e.* 803 K. Therefore the reactor cannot be operated beyond 213.91 bar, although the reactor system is capable of autothermic operation greater than 213.91 bar. Increase in pressure provides more flexibility in operation and NH_3_ production than decrease in pressure, but at the expense of more H_2_ consumption, see Table 3.

**Fig. 4:**
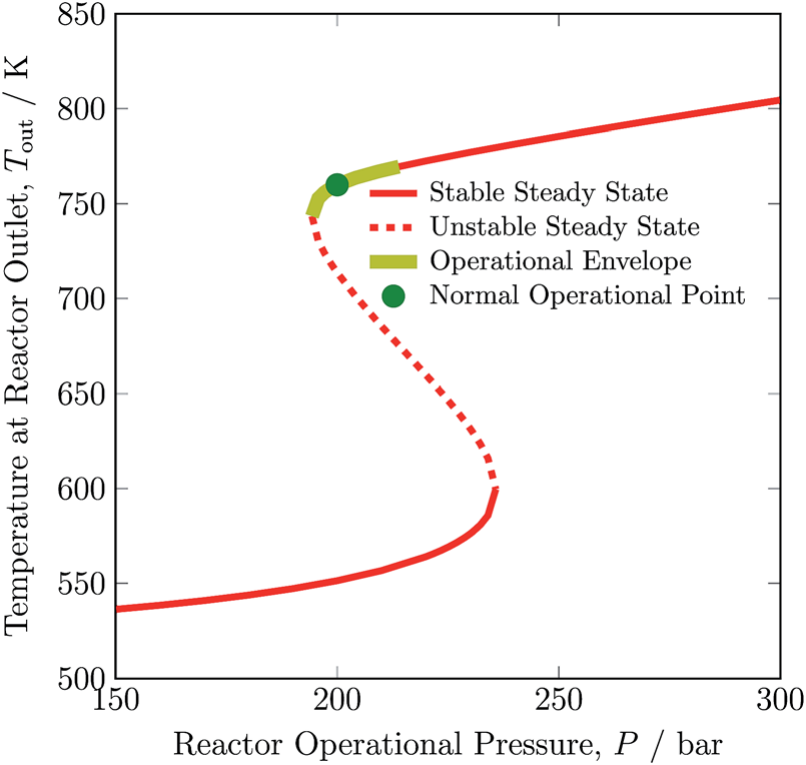
Steady state characteristics of the reactor system for outlet temperature *versus* operational pressure of the reactor system.

The pressure dependence of the outlet temperature is given in Fig. 4. The stable steady state points are covered by the solid line and unstable steady state points by dotted line. The stable operational envelope for pressure is 194.32 to 213.91 bar. Decreasing the inlet temperature at bed 1 or pressure within the reactor system below *ca.* 663 K or 194.32 bar leads to the reactor system shutdown, and increasing inlet temperature at bed 1 or reactor pressure above 679 K or 213.91 bar results at catalyst bed 1 in an exit gas temperature greater than 803 K. In the given pressure range, multiple states are possible and due to this multiplicity the branch switching is also possible. The upper branch is desired for stable steady state operation.

**Fig. 5:**
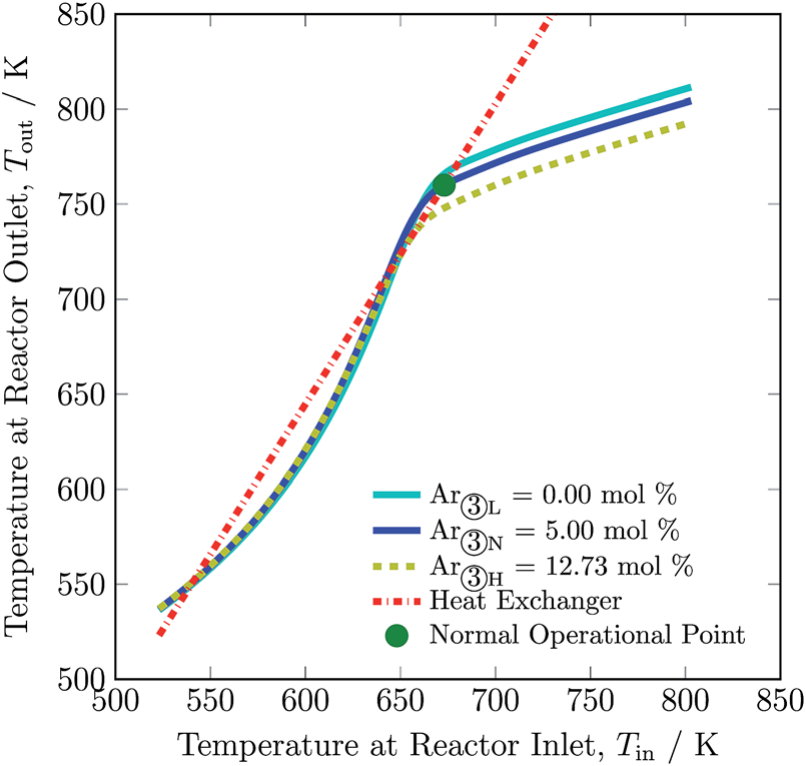
Steady-state characteristics of the reactor system for low (L), normal (N) and high (H) argon (inert gas) concentrations in feed ③ of the reactor system.

The dependence on the stable operating range of the autothermic reactor system on the inert gas concentration in feed is shown in Fig. 5. The exit gas temperature of the reactor system decreases by 30 K, *i.e.* from 760 to 730 K with addition of inert gas in the feed. Temperature of the exit gas increases to *ca.* 770 K with removal of inert gas in the feed, see Table S4, ESI.† The underlying reason is that reactant concentration decreases or increases with addition or removal of inert gas in the feed, respectively. Furthermore, as can be evident from Table 3, with increase and decrease in inert gas concentration in feed, a H_2_ intake decreases and increases in feed by 36.14% and 15.00%, respectively. A maximum operating envelope of 0 to 12.73 mol% inert species is identified. Here, 0 mol% of inert gas means zero purging of gas from recycle stream and fresh stream ① consist of H_2_ and N_2_ only. Inert gas higher than 12.73 mol% is not suitable for autothermic operation of the reactor system, as the heat of removal will be greater than the heat produced by ammonia synthesis reaction.

**Fig. 6:**
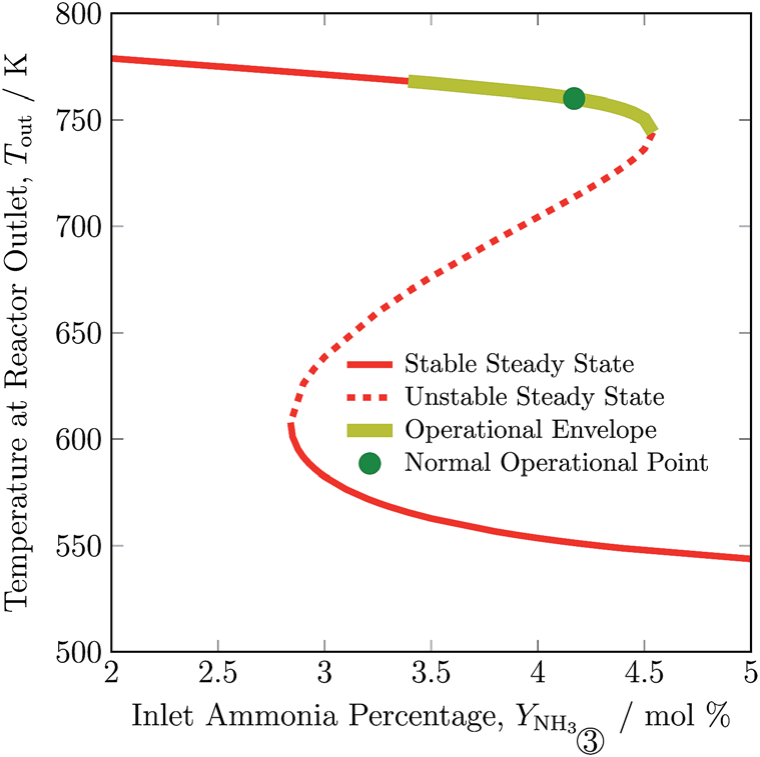
Steady state characteristics of the reactor system for outlet temperature *versus* ammonia concentration in feed ③ of the reactor system.

In Fig. 6, outlet temperature *versus* ammonia concentration in the feed for the reactor system (stream ③) is shown. The reverse S-shaped curve presents up to three steady state points in the range of 2.84 to 4.53 mol% ammonia concentration in feed. The desired operational envelope for ammonia concentration in the feed is quite narrow with 3.39 to 4.53 mol%. The switching of the branch above 4.53 NH_3_ mol% in feed results in reactor operation instability, and operating below 3.39 NH_3_ mol% results in temperature higher than the catalyst sustainability limit in catalyst bed 1, see Table S4, ESI.† A decrease in ammonia concentration in the reactor feed results in higher outlet temperature and higher reactants conversion by 8 K and 1%, respectively from normal operation. The load on the recycle stream is reduced slightly by 1.5%, at the expense of 6% more hydrogen consumption, also see Table 3. Whereas, with an increase in ammonia concentration in the reactor system intake, reactants composition decreases, and results in lower conversion and temperature rise in all catalyst beds.

**Fig. 7:**
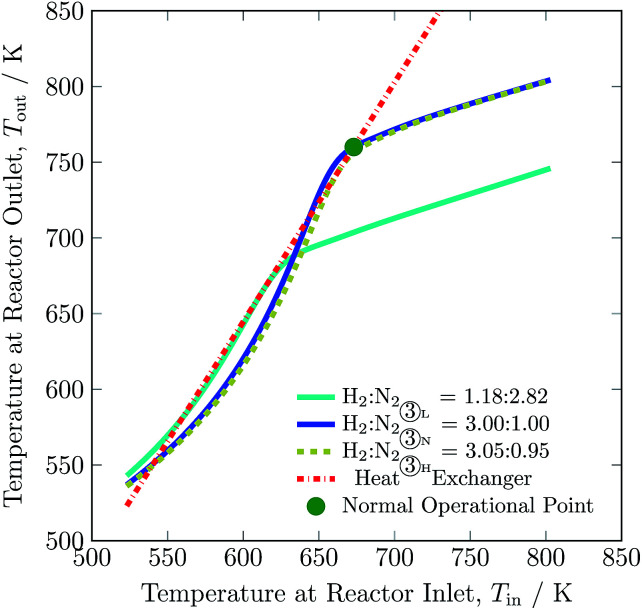
Steady-state characteristics of the reactor system for low (L), normal (N) and high (H) H_2_-to-N_2_ ratios in feed ③ of the reactor system.

The operational envelope for the H_2_-to-N_2_ ratio is quite wide for autothermal operation of the reactor system, which is evident from Fig. 7. The reactor can be operated for H_2_-to-N_2_ ratios between 1.18 : 2.82 and 3.05 : 0.95. However, operating the reactor under a non-stoichiometric ratio noticeably reduces H_2_ intake and increases the recycle load, see Table 3. For the reactor system operation under a non-stoichiometric ratio of reactants, the feed stream ① composition also varies from the nominal value, and new compositions are calculated by using eqn S30 to S32, see ESI.† The reactor at H_2_-to-N_2_ ratio of 1.18 to 2.82 (H_2_ is limiting reactant) and 3.05 to 0.95 (N_2_ is limiting reactant) results in *ca.* 37.5 and 22% of H_2_ conversion, and *ca.* 5 and 23.5% of N_2_ conversion, respectively, compare to *ca.* 24.5% of reactants for normal operation. Also, it should be noted that the reactor temperature decreases by up to 90 K with decrease in H_2_-to-N_2_ ratio and enhances limited reactant conversion, see Table S4, ESI.† The operation of the reactor system at a ratio other than 3 mol of H_2_ to 1 mol of N_2_ reduces NH_3_ production. But the low H_2_-to-N_2_ ratio, which corresponds to a lower hydrogen intake, is still beneficial during renewable power, *i.e.* hydrogen production outage for small period of time, as it will not let the ammonia synthesis reactor blow out. As such, the H_2_-to-N_2_ ratio may be a major manipulable for renewable energy availability based control of such plants.

**Fig. 8:**
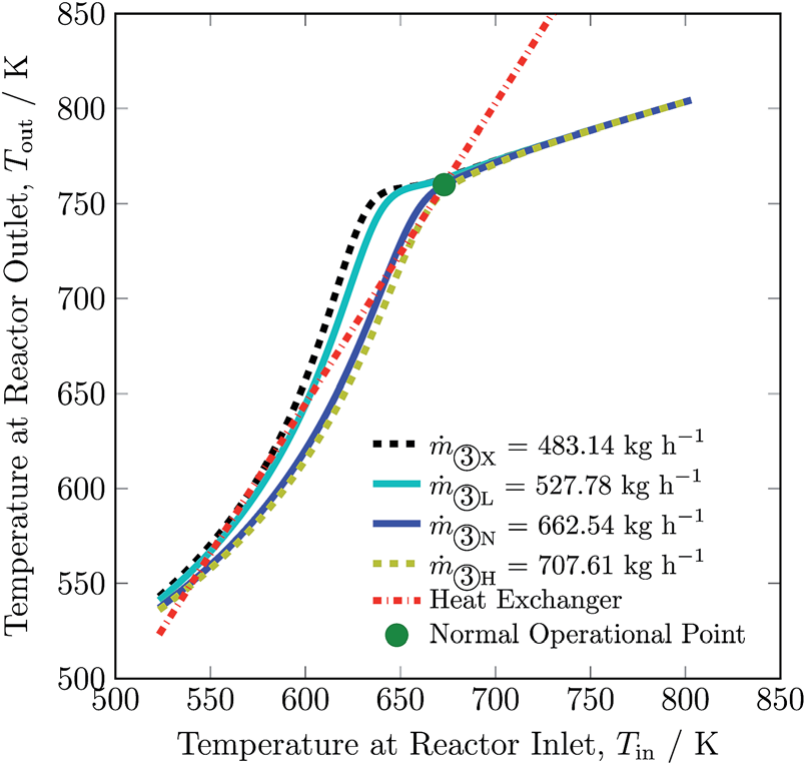
Steady-state characteristics of the reactor system for lowest (X), low (L), normal (N) and high (H) total feed ③ flow rates of the reactor system.

To adjust for fluctuation of renewables, total feed flow inlet may be adjusted. The maximum and minimum total feed flow rates are 707.61 to 527.78 kg h^−1^ respectively, with corresponding ammonia productions of 116.13 and 100.60 kg h^−1^. The change in total feed flow rate is realised by a proportional change in quenches. A decrease in total flow rate results in a decline in the hydrogen intake by *ca.* 16% and in recycle load by *ca.* 16%. On the other hand, significant increase in total flow rate was not possible, and therefore not much change in hydrogen intake and recycle load occurred, see Table 3. The exit temperature (see Fig. 8) and overall conversion of the reactor remains higher for flow rates below the normal total feed flow rate and *vice versa*, see also Table S4, ESI.† This is due to the fact that the reaction reaches equilibrium conditions well before exiting from bed 3 at lower flow rates. Whereas, with increase in flow rate, the space velocity also increases and it results in lower rate of reaction. Like for other process variables, the operating envelope for total feed flow rate also lies inside the multiplicity region, and it is again limited by stability of the reactor system and maximum catalyst temperature in bed 1.

**Fig. 9:**
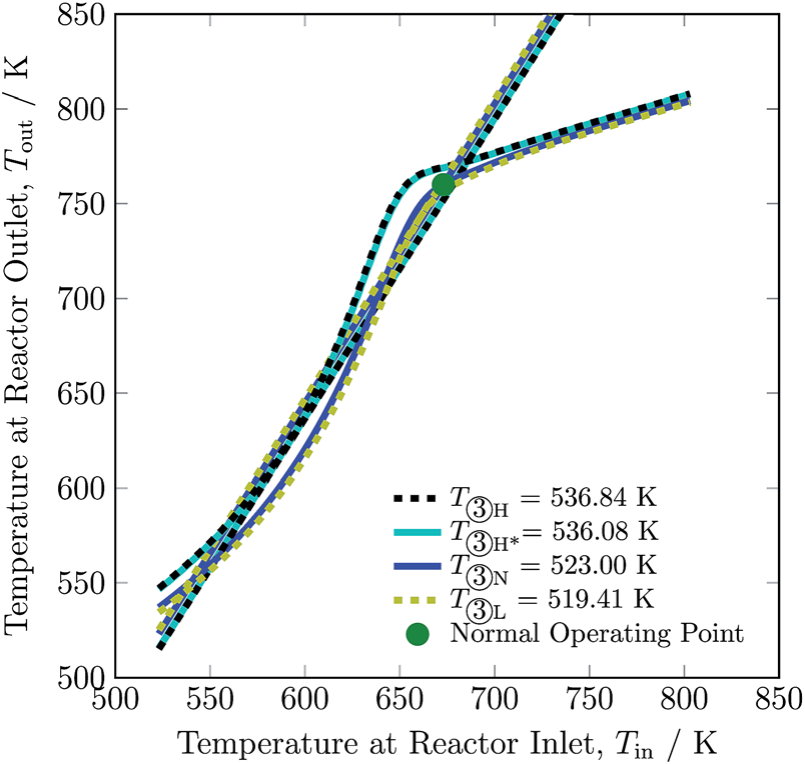
Steady-state characteristics of the reactor system for high (one (H) and two intersections (H*)), normal (N) and low (L) feed ③ temperatures of the reactor system.

Changing the feed temperature entering the reactor system changes not only the heat production curve but also the heat removal line. The feed temperature influences the location of both the curve and the line in the opposite direction: with the increase in feed temperature, the heat production curve moves upwards, while the heat removal line moves downwards, as can be seen in Fig. 9. This distinguishes feed temperature from the other investigated process variables; with changes in feed temperature, the *y*-intercept of heat removal curve also changes, see eqn (2). The operating envelope for the feed temperature is between 519.41 and 536.84 K, where from 519.41 to 536.08 K lies inside the multiplicity region, and above 536.08 K the heat production curve intersects the heat removal line at only one point. The minimum and maximum limit of feed temperature is set due to stability of the reactor system and maximum temperature reached in catalyst bed 1, respectively. Operation of the reactor system at conditions other than normal feed temperature *i.e.* 523 K, reduces H_2_ intake up to *ca.* 8% and NH_3_ production up to *ca.* 8% at the expense of a slight increase of recycle load up to *ca.* 2%, see Table 3. Overall, change in the feed temperature results in a decline in conversion from normal operation, see Table S4.† Whereas, it can be seen that for higher feed temperature, conversion in bed 1 and 2 is higher from normal operation, but conversion in bed 3 is lower, which is attributed to higher temperature operation *i.e.* equilibrium is approached before exit of bed 3.

After comparing results for process variables from Fig. 3 to 9, Tables 3 and S4 (ESI†) we conclude that reducing H_2_-to-N_2_ ratio, increasing inert gas concentration and decreasing feed flow rate have the most potential to reduce the H_2_ consumption by up to *ca.* 67%, 36% and 16%, respectively. This decrease in H_2_ intake comes along with variations in recycle load; with H_2_-to-N_2_ ratio reduction and inert gas concentration increase, the recycle load increases by 17% and 9%, respectively and along with decrease in feed flow rate the recycle load also decreases. Among the six process variables, inert gas concentration in the feed provides the maximum operational flexibility, almost increasing by 255% from the normal value, and without inert gas in the synthesis loop, H_2_ consumption increases by 15%. The other three process variables barely impact H_2_ consumption (below 10%) and recycle load (below 3%), see Table 3. The higher temperature operational limit of 803 K is approached in catalyst bed 1 at a lower boundary of NH_3_ and feed flow rate, and at an upper boundary of pressure and feed temperature.

## 4 Conclusions and outlook

This work presented a systematic analysis of the operating and production flexibility of a Haber–Bosch ammonia reactor. From the results, it can be concluded that the autothermic reactor is viable for power-to-ammonia process, as it can be operated for a wide range of process variables while maintaining operational, hydrogen feed intake and ammonia production flexibilities. Operating outside these boundaries leads to the shutdown of reactor system autothermic operation or damage to the catalyst due to overheating. Among the six process variables, H_2_-to-N_2_ ratio and inert gas concentration in the reactor system feed provide the most flexibilities with up to *ca.* 67% decrease in H_2_ intake. This state may be advantageous to prevent the production plant from shutting down during phases of low availability of the H_2_ produced from the renewables. Further, it can be noted that changes in H_2_-to-N_2_ ratio and feed temperature from the nominal operational values result in a decline in hydrogen intake and ammonia production, causing the load on recycle stream to increase, whereas higher temperature operational limit is always reached in the catalyst bed 1. This study showed that despite present Haber–Bosch reactors being operated only at their optimum, the reactor system is feasible to operate over a wide load range, and is thus attractive for power-to-ammonia applications.

In this work, heat losses to the surroundings are ignored. For smaller scale plants and very low mass feed flow rate, these losses might be noticeable and influence operating envelope. With consideration of design and construction specifications, along with site selection and environmental conditions, heat losses can be within the scope of future work. Furthermore, with consideration of design and operation limitations imposed by the overall synthesis loop, the impact of the work can be enhanced. Further improvements may be done by widening the operating envelope by jointly regulating various process variables, by disproportionately changing the flow rate of quenches and by using catalyst with higher maximum temperature in bed 1. Also, future studies may compare various ammonia synthesis reactor systems for operational and production flexibilities.

## Conflicts of interest

There are no conflicts to declare.

## Nomenclature

### List of symbols

a
: Activity/—
*C*_p_
: Specific heat capacity/kJ kmol^−1^ K^−1^
Δ*H*: Heat of reaction/kJ kmol^−1^
K
: Equilibrium constant/bar^−2^
k
: Reaction rate constant/kmol m^−3^ h^−1^
ṁ
: Mass flow rate/kg h^−1^
ṅ
: Molecular flow rate/kmol h^−1^
*R*_NH_3__
: Rate of reaction/kmol m^−3^ h^−1^
T
: Temperature/K
V
: Volume of catalyst bed/m^3^
X
: Conversion of reactant/—
Y
: Concentration/mol%

### Greek symbols

α
: Constant/0.5
ν
: Stoichiometric coefficient/—
ε
: Heat exchanger effectiveness/—

### Subscripts

2: Reverse reaction
*b*
: Catalyst bed
H: High
in: Inlet
L: Low
*m*
: Mixer
N: Normal
out: Outlet
*q*
: Quench stream
r: Reactant
s: Shell side
t: Tube side
X: Extreme: highest or lowest

## Supplementary Material

RA-008-C8RA06821F-s001
