# Supplementary material for: Operating envelope of Haber–Bosch process design for power-to-ammonia
Source: RSC Adv. 2018 Oct 11;8(61):34926–36. doi: 10.1039/c8ra06821f (PMC9087324; doi:10.1039/c8ra06821f)
Supplement: RA-008-C8RA06821F-s001 [file RA-008-C8RA06821F-s001.pdf]

Electronic Supplementary Material (ESI) for RSC Advances.
This journal is © The Royal Society of Chemistry 2018

RSC Advances

ELECTRONIC SUPPLEMENTARY INFORMATION

Operating Envelope of Haber-Bosch Process Design for Power-to-Ammonia[‡]

Izzat Iqbal Cheema[abc] and Ulrike Krewer[*a]

S.1 Heat Exchanger

For shell and tube heat exchanger, effectiveness ε is calculated as:[1]

$$\varepsilon = \frac{2}{1+C^* + \sqrt{1+C^{*2}}\,\frac{1+\exp[-\mathrm{NTU}\sqrt{1+C^{*2}}]}{1-\exp[-\mathrm{NTU}\sqrt{1+C^{*2}}]}} \tag{S1}$$

where NTU is number of transfer units,

$$\mathrm{NTU} = \frac{UA_{\mathrm{HE}}}{(\dot{m}C_p)_{\mathrm{min}}} \tag{S2}$$

and

$$C^* = \frac{(\dot{m}C_p)_{\mathrm{min}}}{(\dot{m}C_p)_{\mathrm{max}}} \tag{S3}$$

By solving equations (2, S1 to S3), we obtain heat exchanger effectiveness ε, heat capacity ratio C^*, heat exchanger surface area A_{HE} and number of transfer units (NTU) for 50 W m^{-2} K^{-1} overall heat transfer coefficient U[2], see table S1.

Table S1 Heat exchanger of reactor system

ε / –	C^* / –	NTU / –	A_{HE} / m^2
0.6329	0.5734	1.5984	7.0627

S.2 Catalyst Bed

For solving the equations 3 to 5, constants and parameters are required, and they are calculated as follows:

Mole Fraction of Components

Mole fractions y of components N_2, H_2, NH_3 and Ar are calculated by using a material balance with conversion X, inlet molar fraction y_{in} of the components and stoichiometric coefficients ν of the components and reactants $r \in \{N_2, H_2\}$ as follows:

$$y_{\mathrm{N_2}} = \frac{y_{\mathrm{N_2,\,in}} - \frac{\nu_{\mathrm{N_2}}}{\nu_r} X_r y_{r_{\mathrm{in}}}}{1 - \frac{\nu_{\mathrm{NH_3}}}{\nu_r} X_r y_{r_{\mathrm{in}}}} \tag{S4}$$

$$y_{\mathrm{H_2}} = \frac{y_{\mathrm{H_2,\,in}} - \frac{\nu_{\mathrm{H_2}}}{\nu_r} X_r y_{r_{\mathrm{in}}}}{1 - \frac{\nu_{\mathrm{NH_3}}}{\nu_r} X_r y_{r_{\mathrm{in}}}} \tag{S5}$$

$$y_{\mathrm{NH_3}} = \frac{y_{\mathrm{NH_3,\,in}} + \frac{\nu_{\mathrm{NH_3}}}{\nu_r} X_r y_{r_{\mathrm{in}}}}{1 - \frac{\nu_{\mathrm{NH_3}}}{\nu_r} X_r y_{r_{\mathrm{in}}}} \tag{S6}$$

$$y_{\mathrm{Ar}} = \frac{y_{\mathrm{Ar_{in}}}}{1 - \frac{\nu_{\mathrm{NH_3}}}{\nu_r} X_r y_{r_{\mathrm{in}}}} \tag{S7}$$

Activity

Activity of component a_c is defined as ratio between the fugacity of components $c \in \{N_2, H_2, NH_3\}$ at particular arbitrarily chosen state f_c to the fugacity of pure component $f_c{}^*$ at pressure 1 bar and temperature equal to system temperature and is calculated as:

$$a_c = \frac{f_c}{f_c{}^*} = y_c f_c{}^o = y_c \phi_c P \tag{S8}$$

where $f_c{}^o$ is the fugacity at temperature T and pressure P of the system, y_c molar fraction of components and ϕ_c is the fugacity coefficient of components. For nitrogen[3,4], hydrogen[3,5] and ammonia[3,4], the respective fugacity coefficients ϕ_c are as follows:

$$\phi_{\mathrm{N_2}} = 0.93431737 + 0.3101804 \times 10^{-3}\, T + 0.295895 \times 10^{-3}\, P - 0.270729 \times 10^{-6}\, T^2 + 0.4775207 \times 10^{-6}\, P^2 \tag{S9}$$

$$\phi_{\mathrm{H_2}} = \exp\{\mathrm{e}^{(-3.8402\ T^{0.125}+0.541)} P - \mathrm{e}^{(-0.1263\ T^{0.5}-15.980)} P^2 + 300\ [\mathrm{e}^{(-0.011901\ T-5.941)}]\ (\mathrm{e}^{-P/300}-1)\} \tag{S10}$$

$$\phi_{\mathrm{NH_3}} = 0.1438996 + 0.2028538 \times 10^{-2}\, T - 0.4487672 \times 10^{-3}\, P - 0.1142945 \times 10^{-5} \times T^2 + 0.2761216 \times 10^{-6} P^2 \tag{S11}$$

[a] Institute of Energy and Process Systems Engineering, TU Braunschweig, Braunschweig 38106, Germany. Fax: +49 531 3915932; Tel: +49 531 3913030; E-mail: u.krewer@tu-braunschweig.de
[b] International Max Planck Research School for Advanced Methods in Process and Systems Engineering, Magdeburg 39106, Germany.
[c] Department of Chemical, Polymer and Composite Materials Engineering, University of Engineering and Technology, Lahore (KSK - Campus) 39021, Pakistan.
‡ Full paper available: See DOI: 10.1039/xxxxxxxxxx/

Reaction Rate Constant

The reaction rate constant is expressed by Arrhenius equation as a function of temperature:

$$k = k_0\ \mathrm{e}^{-E_\mathrm{a}/RT} \tag{S12}$$

where k is reaction rate constant, k_0 is frequency factor and E_a activation energy for ammonia decomposition, see table S2 for values.

Table S2 Catalyst properties [6]

α	k_0 / kmol m^{-3}	E_a / kJ kmol^{-1}
0.5	8.8490×10^{14}	1.7056×10^{5}

Equilibrium Constant

The equilibrium constant is calculated using the equation developed by Gillespie and Beattie: [7]

$$\log K = -2.691122\ \log T - 5.519265 \times 10^{-5} T + 1.848863 \times 10^{-7} T^2 + \frac{2001.6}{T} + 2.67899 \tag{S13}$$

At equilibrium, forward and reverse rates of reaction will be equal, i.e. $R_{\mathrm{NH_3}} = 0$, and at equilibrium for $\alpha = 0.5$, equation (5) reduces to:

$$K^2 = \frac{a^2{}_{\mathrm{NH_3}}}{a_{\mathrm{N_2}} a^3{}_{\mathrm{H_2}}} \tag{S14}$$

where K is the equilibrium constant. For the equilibrium (EQ) line, equation (S13) and (S14) are solved simultaneously for equilibrium conversion X_{EQ} and temperature T_{EQ}.

Specific Heat Capacity

The specific heat capacities C_{p_c} are expressed as polynomials in T; the pressure dependence for real gases $c \in \{\mathrm{N_2}, \mathrm{H_2}, \mathrm{Ar}\}$ is negligible and calculated as equation (S15) using table S3.

$$C_{p_c} = 4.184\ (A + B\ T + C\ T^2 + D\ T^3) \tag{S15}$$

Table S3 Coefficients of C_p polynomial for equation (S15) [8]

Component	A	$B \times 10^{-2}$	$C \times 10^{-5}$	$D \times 10^{-9}$
N_2	6.903	−0.03753	0.1930	−0.6861
H_2	6.952	−0.04576	0.09563	−0.2079
Ar	4.9675			

For NH_3, pressure dependence is expressed by coefficients of polynomials as linear function of P by Shah. [9] Equation (S16) is taken from data at two pressures, 200 and 600 atm.

$$C_{p_{\mathrm{NH_3}}} = 4.184\ \{6.5846 - 0.61251 \times 10^{-2}\ T + 0.23663 \times 10^{-5}\ T^2 - 1.5981 \times 10^{-9}\ T^3 + [96.1678 - 0.067571\ P + (-0.2225 + 1.6847 \times 10^{-4}\ P)\ T + (1.289 \times 10^{-4} - 1.0095 \times 10^{-7}\ P)\ T^2]\} \tag{S16}$$

The specific heat capacity for gas mixture consisting of components $c \in \{\mathrm{N_2}, \mathrm{H_2}, \mathrm{Ar}, \mathrm{NH_3}\}$ in catalyst bed C_{p_b} is:

$$C_{p_b} = (\sum_{c=1}^{n} y_{c_b} C_{p_{c_b}})/M_{\mathrm{mix}_b} \tag{S17}$$

where M_{mix_b} is the average molecular weight of gas mixture.

Heat of Reaction

The expression for heat of reaction ΔH is obtained in a similar manner to specific heat of NH_3 (equation S16) by accommodating pressure dependency of coefficients. [10]

$$\Delta H = 4.184\ [-(0.54526 + 846.609\ T^{-1} + 459.734 \times 10^6\ T^{-3})P - 5.34685\ T - 0.2525 \times 10^{-3}\ T^2 + 1.69197 \times 10^{-6}\ T^3 - 9157.09] \tag{S18}$$

S.3 Ammonia Synthesis Loop

The material balance for ammonia synthesis loop (figure S1) is defined by equations S19 to S33. The components material balance around the splitter is given as follows:

$$\dot{m}_{c\,②} = \dot{m}_{c\,⑤} - \dot{m}_{c\,⑥} \tag{S19}$$

where components $c \in \{\mathrm{N_2}, \mathrm{H_2}, \mathrm{NH_3}, \mathrm{Ar}\}$ concentration in streams ②, ⑤ and ⑥ are same, i.e. $x_{c\,②} = x_{c\,⑤} = x_{c\,⑥}$ and $y_{c\,②} = y_{c\,⑤} = y_{c\,⑥}$, as purging is carried out by splitting stream ⑤ into two streams. Therefore,

$$\dot{m}_{c\,②} = (1 - p)\ \dot{m}_{c\,⑤} \tag{S20}$$

where p is defined as ratio between stream ⑥ and stream ⑤:

$$p = \frac{\dot{m}_{⑥}}{\dot{m}_{⑤}} = \frac{\dot{m}_{c\,⑥}}{\dot{m}_{c\,⑤}} \tag{S21}$$

The material balance around the separator for 100 % pure NH_3 net product ($y_{\mathrm{NH_3}⑦} = x_{\mathrm{NH_3}⑦} = 1$) and components $c \in \{\mathrm{N_2}, \mathrm{H_2}, \mathrm{Ar}\}$ is given as follows:

$$\dot{m}_{c\,④} = \dot{m}_{c\,⑤} \tag{S22}$$

The material balance for components around the mixer is stated as follows:

$$\dot{m}_{c\,③} = \dot{m}_{c\,②} + \dot{m}_{c\,①} \tag{S23}$$

where $\dot{m}$ is mass flow rate for components $c \in \{N_2, H_2, NH_3, Ar\}$ in streams ①, ② and ③. For NH_3 free fresh feed ($y_{NH_3①} = x_{NH_3①} = 0$), equation S23 reduces and resulted as follows:

$$\dot{m}_{NH_3③} = \dot{m}_{NH_3②} \tag{S24}$$

Substituting equation S24 into S20 results in the following:

$$\dot{m}_{NH_3③} = (1 - p)\, \dot{m}_{NH_3⑤} \tag{S25}$$

In addition, the mass flow rate of components $c \in \{N_2, H_2, NH_3, Ar\}$ in streams Ⓢ $\in \{1,2,3,4,5,6,7\}$ of synthesis loop can be expressed as follows:

$$\dot{m}_{c\,Ⓢ} = x_{c\,Ⓢ}\, \dot{m}_{Ⓢ} \tag{S26}$$

where x is components mass fraction.
By combining equations S19, S21, S23, S22 and S26, we get generalised equation for components $c \in \{N_2, H_2, Ar\}$:

$$\dot{m}_{c\,③} - \dot{m}_{c\,④} + p\,\dot{m}_{c\,④} - x_{c\,①}\dot{m}_{①} = 0 \tag{S27}$$

For fresh feed ① consisting of N_2, H_2 and Ar, where N_2 is supplied along with 2 mol % (0.285 wt %) of Ar, see section 2.2. Thus,

$$x_{Ar①} = 0.0285 x_{N_2①} \tag{S28}$$

$$x_{H_2①} = 1 - x_{N_2①} - x_{Ar①} \tag{S29}$$

By inserting equations S28 and S29 into equation S27 for Ar and H_2. The mass balance of components $c \in \{N_2, H_2, Ar\}$ for overall synthesis loop from equation S27 expressed as follows:

$$\dot{m}_{N_2\,③} - \dot{m}_{N_2\,④} + p\,\dot{m}_{N_2\,④} - x_{N_2\,①}\dot{m}_{①} = 0 \tag{S30}$$

$$\dot{m}_{H_2\,③} - \dot{m}_{H_2\,④} + p\,\dot{m}_{H_2\,④} - (1 - 1.0285 x_{N_2①})\dot{m}_{①} = 0 \tag{S31}$$

$$\dot{m}_{Ar\,③} - \dot{m}_{Ar\,④} + p\,\dot{m}_{Ar\,④} - 0.0285 x_{N_2①}\dot{m}_{①} = 0 \tag{S32}$$

By solving equations S30, S31 and S32 simultaneously, p, $x_{N_2①}$ and $\dot{m}_{①}$ can be expressed.
The overall total material balance for ammonia synthesis loop is given as follows:

$$\dot{m}_{①} = \dot{m}_{⑥} + \dot{m}_{⑦} \tag{S33}$$

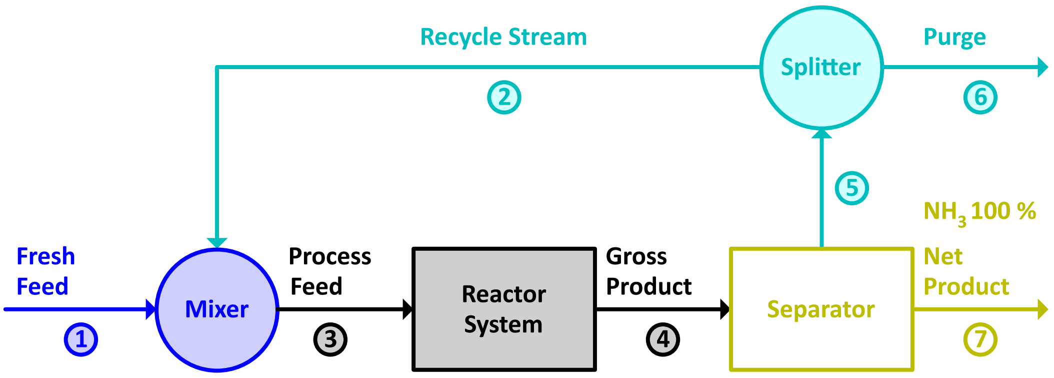

Fig. S1 Block diagram of ammonia synthesis loop

S.4 Simulation Data

Table S4 Temperatures and conversions of the reactor system for normal operation and operation at the boundary of the operating envelope

Process Variables	Operating Envelope			Bed-1 in	Bed-1 out	Bed-2 in	Bed-2 out	Bed-3 in	Bed-3 out
Normal Operation (See table 1)			T / K	673.00	773.00	673.00	772.93	673.00	760.01
			X_{H_2} / %	0.00	11.31	7.50	19.19	14.06	24.68
			X_{N_2} / %	0.00	11.31	7.50	19.19	14.06	24.68
			N_2 / mol %	22.71	21.23	21.74	20.10	20.84	19.26
			H_2 / mol %	68.12	63.69	65.24	60.31	62.54	57.79
			NH_3 / mol %	4.17	9.81	7.84	14.11	11.28	17.32
			Ar / mol %	5.00	5.27	5.18	5.48	5.34	5.63
P / bar	194.32	Low	T / K	662.50	740.50	655.63	739.38	657.12	743.42
			X_{H_2} / %	0.00	8.97	5.95	15.88	11.64	22.17
	213.91	High	T / K	679.05	803.00	688.81	792.20	682.48	769.56
			X_{H_2} / %	0.00	13.78	9.13	21.14	15.49	26.09
Y_{Ar} ③ / mol %	0.00	Low	T / K	679.23	785.50	680.55	784.75	680.19	769.86
			X_{H_2} / %	0.00	11.51	7.62	19.26	14.11	24.53
	12.73	High	T / K	654.04	724.85	646.50	723.56	648.50	730.05
			X_{H_2} / %	0.00	8.74	5.79	15.64	11.46	22.21
Y_{NH_3} ③ / mol %	3.39	Low	T / K	678.08	803.00	688.46	790.56	682.17	768.04
			X_{H_2} / %	0.00	13.83	9.16	20.98	15.38	25.78
	4.53	High	T / K	662.96	739.98	655.42	739.57	656.93	744.14
			X_{H_2} / %	0.00	8.89	5.89	15.89	11.65	22.32
H_2:N_2 ③ / mol of H_2 : mol of N_2	1.18:2.82	Low	T / K	613.89	654.25	606.10	653.13	610.82	666.61
			X_{H_2} / %	0.00	12.41	8.22	23.87	17.34	37.46
			X_{N_2} / %	0.00	1.74	1.15	3.18	2.33	4.74
	3.05:0.95	High	T / K	663.71	743.30	657.23	742.38	658.67	745.33
			X_{H_2} / %	0.00	8.98	5.95	15.84	11.61	21.98
			X_{N_2} / %	0.00	9.61	6.37	17.00	12.46	23.65
$\dot{m}$③ / kg h^{-1}	527.78	Low	T / K	675.49	803.00	687.54	787.20	677.98	763.95
			X_{H_2} / %	0.00	14.20	9.41	21.06	15.44	25.97
	707.61	High	T / K	664.02	743.10	657.24	741.97	658.74	745.81
			X_{H_2} / %	0.00	9.08	6.01	16.04	11.75	22.36
T③ / K	519.41	Low	T / K	661.08	742.07	654.67	741.03	655.66	743.25
			X_{H_2} / %	0.00	9.30	6.16	16.39	12.01	22.72
	536.84	High	T / K	686.02	803.00	696.22	792.28	692.55	772.56
			X_{H_2} / %	0.00	13.05	8.65	19.82	14.52	24.22

Nomenclature

List of Symbols

A	Constant for heat capacity / -
a	Activity / -
A_{HE}	Surface area for heat transfer / m^2
B	Constant for heat capacity / -
C	Constant for heat capacity / -
C^*	Heat capacity rate ratio / -
C_p	Specific heat capacity / kJ $kmol^{-1}$ K^{-1}
D	Constant for heat capacity / -
E_{a}	Activation energy / kJ $kmol^{-1}$
f	Fugacity / -
K	Equilibrium constant / bar^{-2}
k	Reaction rate constant / kmol m^{-3} h^{-1}
k_0	Frequency factor / kmol m^{-3} h^{-1}
M	Molecular weight / kg $kmol^{-1}$
$\dot{m}$	Mass flow rate / kg h^{-1}
NTU	Number of transfer units / -
P	Pressure / bar
R	Universal gas constant / 8.314 kJ $kmol^{-1}$ K^{-1}
$R_{\mathrm{NH_3}}$	Rate of reaction / kmol m^{-3} h^{-1}
U	Overall heat transfer coefficient / W m^{-2} K^{-1}
x	Mass fraction / -
y	Mole fraction / -
Y	Concentration / mol %

Greek Symbols

α	Constant / 0.5
ν	Stoichiometric coefficient / -
ϕ	Fugacity coefficient / -
ε	Heat exchanger effectiveness / -

Superscripts

$*$	At a particular arbitrarily chosen standard state
o	At temperature and pressure of system

Subscripts

2	Reverse reaction
b	Catalyst bed
c	Component
in	Inlet
max	Maximum
min	Minimum
mix	Gas mixture
out	Outlet
r	Reactant
S	Stream
s	Shell side
t	Tube side

References

1 S. Kakaç, H. Liu and A. Pramuanjaroenkij, *Heat Exchangers: Selection, Rating, and Thermal Design*, CRC Press, 3rd edn, 2012.
2 O. Frank, *Chem. Eng.*, 1974, **81**, 126–127.
3 H. W. Cooper, *Hydrocarbon Process.*, 1967, **46**, 159–160.
4 R. H. Newton, *Ind. & Eng. Chem.*, 1935, **27**, 302–306.
5 R. S. Herbert and R. W. David, *Am. J. Sci.*, 1964, **262**, 918–929.
6 D. C. Dyson and J. M. Simon, *Ind. Eng. Chem. Fundam.*, 1968, **7**, 605–610.
7 L. J. Gillespie and J. A. Beattie, *Phys. Rev.*, 1930, **36**, 743.
8 O. A. Hougen and K. M. Watson, *Chemical Process Principles, Part III*, Wiley, New York, NY, 1962, pp. 805–901.
9 M. Shah, *Ind. Eng. Chem.*, 1967, **59**, 72–83.
10 A. T. Mahfouz, S. S. Elshishini and S. S. E. H. Elnashaie, *Modelling, Simulation & Control. B*, 1987, **10**, 1–17.
